# A Multi-Material Flame-Retarding System Based on Expandable Graphite for Glass-Fiber-Reinforced PA6

**DOI:** 10.3390/polym15204100

**Published:** 2023-10-16

**Authors:** Florian Tomiak, Melanie Zitzmann, Dietmar Drummer

**Affiliations:** 1 Institute of Polymer Technology, Friedrich-Alexander-University Erlangen-Nürmberg, Am Weichselgarten 10, 91058 Erlangen, Germany; mezitz@gmx.net (M.Z.);; 2 Bavarian Polymer Institute, Friedrich-Alexander-University Erlangen-Nürnberg, Dr. Mack Strasse 77, 90762 Fuerth, Germany

**Keywords:** PA6, flame retardancy, expandable graphite, aluminum diethylphosphinate, melamine polyphosphate, montmorillonite

## Abstract

A synergistic multi-material flame retardant system based on expandable graphite (EG), aluminum diethylphosphinate (AlPi), melamine polyphosphate (MPP), and montmorillonite (MMT) has been studied in glass-fiber-reinforced polyamide 6 (PA6). Analytical evaluations and fire performances were evaluated using coupled thermogravimetric analysis (TGA) and Fourier-transform infrared spectroscopy (FTIR) as well as cone calorimetry, UL-94 fire testing, and limiting oxygen index (LOI). A combination of EG/AlPi/MPP/MMT has been shown to provide superior flame-retarding properties when integrated at 20 wt.% into glass-fiber-reinforced PA6 (25 wt.%), achieving UL-94 V0 classification and an oxygen index of 32%. Strong residue formation resulted in low heat development overall, with a peak heat release rate (pHRR) of 103 kW/m^2^, a maximum of average heat release rate (MAHRE) of 33 kW/m^2^, and deficient total smoke production (TSP) of 3.8 m^2^. Particularly remarkable was the structural stability of the char residue. The char residue could easily withstand an areal weight of 35 g/cm^2^, showing no visible deformation.

## 1. Introduction

Non-reinforced polyamide 6 (PA6) is known for strong burn dripping behavior, extinguishing the upward burning process in vertical UL-94 burning tests and thus resulting in a V2 classification. The integration of glass fibers changes two essential fire phenomena: (1) Due to an increased melt viscosity, melt dripping is substantially reduced, stabilizing the burning process in vertical UL-94 burning tests. Accordingly, flame spread continues upwards, resulting in a lack of a UL-94 classification. (2) Simultaneously, protruding glass fibers increase the surface area and provide improved thermal conductivity and pyrolysis supply due to the “wicking effect”. Both effects substantially change the physical conditions and accelerate the burning process [1]. Halogenated flame retardant additives (FR) have been proven to work efficiently for glass-fiber-reinforced PA6. However, due to rising environmental requirements and toxicity concerns, halogenated FRs are increasingly substituted [2]. Many publications have been studying phosphorous and nitrogen-containing FR alternatives such as melamine polyphosphate (MPP) [3], melamine cyanurate (MC) [4,5], phosphonate oligomers PSA and POSC [6], aluminum diethyl phosphinate (AlPi) [5,7,8], zinc borate (ZB) [5,9], microencapsulated red phosphorus (RP) [10], and phosphorus oxynitride (PO) [11]. Furthermore, many additives have been found to show superior fire retardant properties in glass fiber when synergistically combined in multi-material systems.

MPP is a commonly used flame retardant additive for glass-fiber-reinforced PA6 and PA6.6 systems. Research has demonstrated that the integration of MPP triggers the formation of a phosphorus-rich intermediate phase on the glass fiber surface. As a result, the limiting oxygen index (LOI) can be increased for PA6 to approximately 29 vol.% (15 wt.% filling degree) and for PA6.6 to 38–40 vol.% (15 wt.% filling degree). The observed difference in flame retardancy performances can be attributed to two possible modes of action. While the incorporation of MPP in PA6.6 initiates strong crosslinking, PA6/MPP combinations instead result in catalytical depolymerization (a decrease in viscosity [12]). Earlier depolymerization affects the flammability behavior in two contrary phenomena. Caprolactam is released at lower temperatures, accelerating the early flame spread since it provides a steady fueling of combustible pyrolysis gases to the flame. On the contrary, a lower fuel supply maintains the low flame growth rate, substantially limiting the heat release rate potential. Both effects reduce and improve fire retardancy properties but differ in efficiency. Additionally, decomposition products often form phosphoric acid, promoting char formation on the polymer’s surface offering insulating characteristics [13].

Metal salts of alkyl phosphinate, such as aluminum diethyl phosphinate (AlPi), have also been shown to provide excellent flame retardancy properties in non-reinforced PA6.6. Two mechanisms are reported: (1) the formation of aluminum phosphate in the condensed phase and (2) the release of diethylphosphinic acid in the gas phase [14]. However, AlPi does not provide a V0 classification in a UL94 test when used as a single FR additive in glass-fiber-reinforced PA6 and PA66. The performance drop can be explained by a capillary-driven effect that accelerates the pyrolysis gas flow, suppressing the flame retardant efficiency of the condensed phase mechanisms [15,16].

Furthermore, synergistic or catalytic effects have been reported when metal salts and polyphosphate compounds, such as MPP and AlPi, are combined. While MPP primarily acts in the gas phase via flame inhibition, combining AlPi and MPP results in a changed reaction path primarily occurring in the condensed phase. The reaction is based on a crosslinking mechanism by forming polyphosphate esters [17]. A synergistic effect of MPP and AlPi has been demonstrated for PA6.6 [18] and PA6 [19].

A well-known strategy to reduce the pHRR or improve the LOI and UL-94 fire testing performance in PA6 recipes is the combined utilization of condensed and gas phase flame retardancy effects [20,21]. Nanofillers have been successfully tested as synergistic additives for a variety of phosphorous FRs. They are predominantly known to improve fire performance via structural char residue enhancement and by providing a labyrinth effect, but can also act catalytically during polymer decomposition [13]. A recent study has reported that a combination of MPP with MMT (nanofiller) can synergistically enhance the flame retardancy properties of GF-reinforced PA6, resulting in an improved V0 UL94 (V) rating [4]. MMT has been shown to reduce the pyrolysis rate through a labyrinth effect as well as residual formation due to clay surface migration. The latter results in a clay layer barrier that improves insulation properties against an external heat source and provides a long-term barrier, hindering the pyrolysis gas supply [20,22]. Other studies have investigated the use of expandable graphite (EG) as an alternative for phosphorus flame retardant additives, which were shown to be very effective in non-reinforced PA6 [23,24] and only somewhat effective in glass-fiber-reinforced PA6 [25]. EG acts physically via the formation of a voluminous, thermally stable residue, providing excellent long-term fire protection properties [23,25]. Due to its solely physical effect, the usage of expandable graphite has been reported for various polymeric systems (e.g., PE [26,27], PP [28,29], PS [30], PVC [31], ABS [32], PA6 [33]).

Recent studies have shown that expandable graphite (EG) can provide a very effective, long-term heat barrier when integrated into GF-reinforced PA6. However, no sufficient UL-94 classification could be reached (V2) [25,34,35]. Within this study, expandable graphite (EG), aluminum diethylphosphinate (AlPi), melamine polyphosphate (MPP), and montmorillonite (MMT) have been investigated as one flame retardant multi-material system for glass-fiber-reinforced PA6. The fundamental concept is based on a combination of a long-term barrier effect (EG), char residue formation, and low pyrolysis rates (AlPi/MPP/MMT), improving flammability and heat development properties. Flammability properties and burning behavior have been investigated using the limiting oxygen index (LOI), UL-94 (V), and cone calorimeter tests. Coupled thermogravimetric analysis (TGA) and Fourier-transform infrared spectroscopy (FTIR) have been used for thermal degradation and chemical analysis. Previous studies that have investigated the flame retardant effect of EG on non-reinforced [23] and reinforced PA6 [25] combinations are referred to as the work that forms the basis for the present investigation.

## 2. Materials and Methods

### 2.1. Materials and Preparation

As the matrix polymer, standard-grade polyamide 6 B27E (PA6) from BASF SE (Ludwigshafen, Germany) was reinforced with glass fibers (GFs) of the type CS7920, provided by Lanxess (Cologne, Germany). The flame retardant additives used were expandable graphite (EG) GHL HT 270 from LUH GmbH (Walluf, Germany), aluminum diethyl phosphinate (AlPi) Exolit 1230 from Clariant AG (Muttenz, Switzerland), melamine polyphosphate (MPP) MP200 from BASF SE (Ludwigshafen, Germany), and organic Montmorillonite MAX CT 4260 from BYK-Chemie GmbH (Wesel, Germany). All materials are listed in Table 1.

The multi-material systems were produced using a co-rotating, twin-screw extruder DSE ZSE HP 27 from Leistritz GmbH (Nuremberg, Germany). Barrel temperatures were controlled between 240 and 220 °C; the screw speed was held constant at 100 rounds per minute (rpm) at a feed rate of 10 kg per hour (kg/h). The multi-material systems containing EG, AlPi, MPP, and MMT were premixed in a universal powder mixer (200 u/min; 15 min) before processing. Two gravimetrical side feeding units were used for glass fibers and the premixed multi-material additive mixture. The strand was drawn off via a water bath chipped to granulate, and dried afterward before further processing. Injection molding was used to mold plate geometries (100 × 100 × 2 and 4 mm^3^), which were then further processed via sawing and milling to generate sample geometries following the individual testing standards. Formulations tested and discussed within this study are listed in Table 2.

### 2.2. Thermal and Gas Analytics

Thermogravimetric analysis (TGA) and coupled Fourier-transform infrared spectrometry (FTIR) were used to analyze changes in the decomposition behavior. TGA measurements were conducted at various heating rates of 2.5, 5, and 10 K/min under a nitrogen atmosphere, using constant temperature ramps between 50 and 800 °C. Therefore, an STA F3 449 Jupiter from Netzsch GmbH (Selb, Germany) was used. Decomposition/pyrolysis gases were transferred from the TGA exhaust to the FTIR gas cell Tensor 2 from Bruker Corp. (Billerica, MA, USA) via a coupled and temperature-controlled transfer line (230 °C). Due to the transfer time between the TGA exhaust and FTIR measurement, a signal delay between TGA and FTIR of about 30 s had to be considered. Activation energies were calculated from non-isothermal TGA measurements using a method suggested by Ozawa and Vyazovkin [36,37,38]. Calculations were performed using an open access calculation tool. The descriptions and download link can be found in [39].

### 2.3. Fire Testing

To study fire retardancy properties, limiting oxygen index (LOI), UL-94, and cone calorimeter (CC) tests were conducted. All testing devices were from Netzsch Taurus Instruments GmbH (Weimar, Germany).

The LOI fire test is particularly useful to track flammability properties occurring from slight formula changes within multi-material flame retardant systems. The testing setup includes a vertically clamped sample (125 × 10 × <10 mm^3^), which is surrounded by a controlled N_2_/O_2_ atmosphere. During the testing routine, a 50 watt propane flame is systematically applied to the sample in a candle-like setup from above. If the sample is ignited, flame propagation is observed, the propagation time and distance are measured, and the N_2_/O_2_ atmosphere contents are systematically adjusted. The resulting key figure “limiting oxygen index” (LOI) represents the lowest atmospheric oxygen content needed to provide either 50 mm downward fire propagation or 3 min of burning time. Tests were conducted following the DIN EN ISO 4589-2 [40] standards.

UL-94 (vertical) fire testing setups are used to study the self-extinguishing properties and fire-dripping behavior of polymeric materials under normal atmospheric conditions. During the test, a 50 watt methane flame is applied from underneath the sample (125 × 13 × <13 mm^3^) following a standardized routine. After the testing flame is removed, burning times and dripping behavior are observed. Testing results are then clustered into three classifications from best to worst: V0, V1, and V2. V0 represents instant self-extinguishing behavior with no burn dripping. V2 classifications allow burn dripping and, compared to V0 classifications, longer burning times. Tests were conducted in accordance with the DIN EN 60695-11-10 [41] standards.

The cone calorimeter testing method is a valuable tool to gain insights into the burning behavior under enforced flaming conditions. During the testing routine, 100 × 100 × < 50 mm^3^ sample geometry is placed underneath the cone heater. Pyrolysis gases are ignited via piloted ignition and the burning process is monitored by tracking the heat development over time until complete combustion. The amount of heat released as well as the heat development characteristics provide important information about the functionality of the flame retardant formulation. Within this study, tests have been conducted using three heater capacities 35, 50, and 65 kW/m^2^ in order to calculate the heat release parameter (HRP). The appropriate literature targeting the correct use and interpretation of important key figures can be found in [42,43,44,45]. The sample geometries used were 100 × 100 × 4 mm^3^. All tests were repeated at least three times, and averaged curves are presented in the results section. Tests were conducted in accordance with the DIN ISO 5660-1 [46] standards.

### 2.4. Char Residue Analysis

Char residue analysis was conducted using a SEM Ultra Plus system from Zeiss (Oberkochen, Germany). Samples were taken via cone calorimeter testing after complete combustion and prepared using platin–palladium.

## 3. Results and Discussion

### 3.1. Thermal Analysis and Evolved Gas Analysis—TGA-FTIR

**Figure 1:**
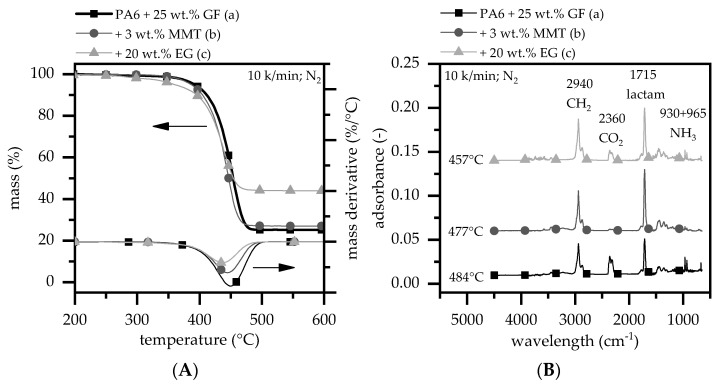
(**A**) TGA analysis measured at a heating rate of 10 K/min under a nitrogen atmosphere. The mass loss tracked over a constant heating rate gives insights about the decomposition process of a polymeric system. (**B**) Corresponding FTIR evolved gas analysis at the DTG (mass derivative) peak temperature.

TGA measurements were taken to evaluate the decomposition behavior of all of the tested formulations. Tests were performed at heating rates of 2.5, 5, and 10 K/min under a nitrogen atmosphere. The results presented in Figure 1 show TGA-FTIR results for a heating rate of 10 K/min without (A) and with in situ FTIR coupling (B).

The TGA analysis of PA6 containing 25 wt.% glass fibers showed a single gravimetrical step with the DTG peak appearing at approximately 451 °C (Figure 1A). Once a temperature of 500 °C was reached, all polymer fractions had evaporated, leaving only the glass fiber residue (25 wt.%). The evolved gases found were caprolactam (with a lactam peak at 1715 cm^−1^ and a fingerprint pattern at 1305, 1352, and 1361 cm^−1^), CO_2_ (at 2360 and 671 cm^−1^), ammonia (at 930 and 965 cm^−1^), CH_2_ groups (at 2940, 2865, and 1140 cm^−1^), and some traces of CO (at 2114 and 2174 cm^−1^) (Figure 1B). These gas phase products have also been found in numerous other studies as part of the PA6 decomposition [47,48,49,50]. When 3 wt.% MMT is integrated, no observable changes in the decomposition onset temperature can be identified (Figure 1A). However, the DTG peak appears to occur slightly earlier at around 445 °C (also found in [51]). This earlier gravimetrical decrease corresponds to a higher concentration of caprolactam in the gas phase (1715 cm^−1^), which can be observed through a relative comparison of the FTIR peak heights between caprolactam and CH_2_ (Figure 1B). Also, a lower amount of CH_3_ and CO_2_ seems to be evaporated, which indicates an additional formation of intermediate aromatic compounds or intermediate bonding with the MMT surface. Since the residual fraction measured at 500 °C is in accordance with the filler integration (GF + MMT) and no changes within the gas phase composition occurred, no alterations of the main decomposition path of PA6 can be observed after the integration of MMT. This effect has also been discussed in other studies [52,53].

When EG is integrated into reinforced PA6, a second decomposition step between 270 and 330 °C can be observed, resulting in a slight gravimetrical decrease of approximately 2.5 wt.%. Once the temperature exceeds 330 °C, the decomposition process accelerates, with a DTG peak appearing at 437 °C. The residual fraction left at 500 °C of 44 ± 1 wt.% is in accordance with the expected range; thus, no additional char formation can be observed. The slightly lower onset temperature for decomposition can be attributed to the expansion mode of EG, causing the sulfuric blowing agent to decompose and gasify. Compared to PA6 GF formulations, FTIR analyses reveal no changes within the gas phase composition. Thus, no alterations of the main decomposition path are triggered by EG integration.

**Figure 2:**
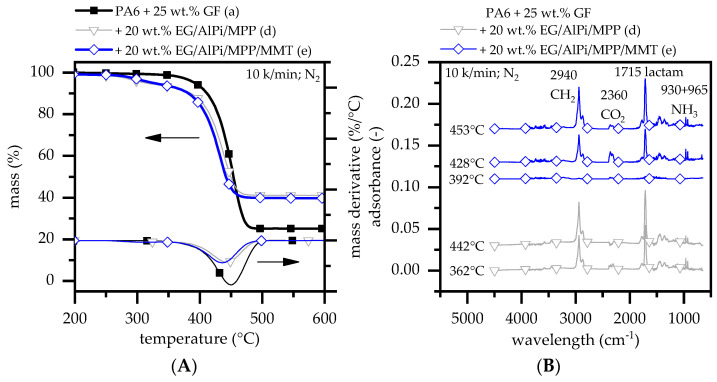
(**A**) TGA analysis measured at a heating rate of 10 K/min under nitrogen atmosphere. The mass loss tracked over a constant heating rate gives insights about the decomposition process of a polymeric system. (**B**) Corresponding FTIR evolved gas analysis.

When integrating EG (15 wt.%), AlPi (3 wt.%), and MPP (2 wt.%) as a multi-material system into glass-fiber-reinforced PA6, two gravimetrical decomposition steps can be observed (Figure 2A). Similar to recipes based on glass-fiber-reinforced PA6 and EG, the initial gravimetrical step occurs between 270 °C and 330 °C marking the start of the EG expansion process. Exceeding 330 °C, the decomposition process accelerates between 400 and 470 °C. The DTG peak occurs at 443 °C, which is slightly higher than that observed for reinforced PA6/EG formulations (437 °C). The residue formation of 41 ± 1 wt.% (500 °C) is in line with expectations. Similar results have been reported in previous studies for non-reinforced PA6 [23,25,35].

When MMT is additionally added into the formulation with EG (15 wt.%), AlPi (2.4 wt.%), MPP (1.6 wt.%), and MMT (1 wt.%) as a multi-material system, only slight changes within the TGA measurements can be observed. The DTG peaks shift slightly to a lower temperature of around 429 °C. The char residue at 600 °C remains within the expected range of 40 ± 1 wt.%. FTIR gas phase analysis also indicates no changes in the gas phase composition for any of the tested multi-material formulations (Figure 2B). However, a shift in the gravimetrical decomposition step toward lower temperatures indicates an accelerated depolymerization effect as well as early evaporation of the EG intercalated blowing agent. All major TGA data are summarized in Table 3.

### 3.2. Burning Behavior—Cone Calorimeter

**Figure 3:**
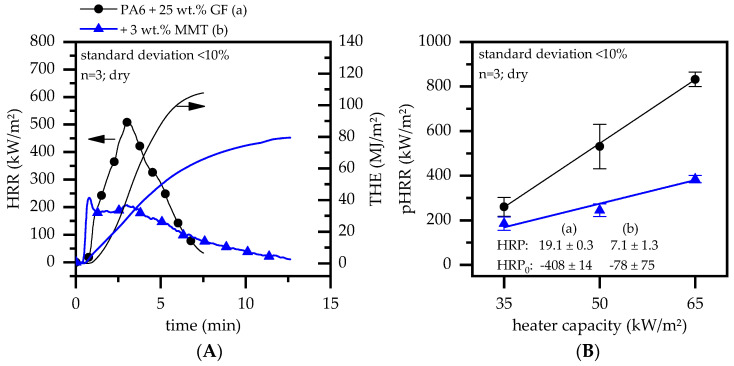
(**A**) Cone calorimeter results measured at 50 kW/m^2^. (**B**) Heat response parameters calculated from the cone calorimeter results at 35, 50, and 65 kW/m^2^.

**Figure 4:**
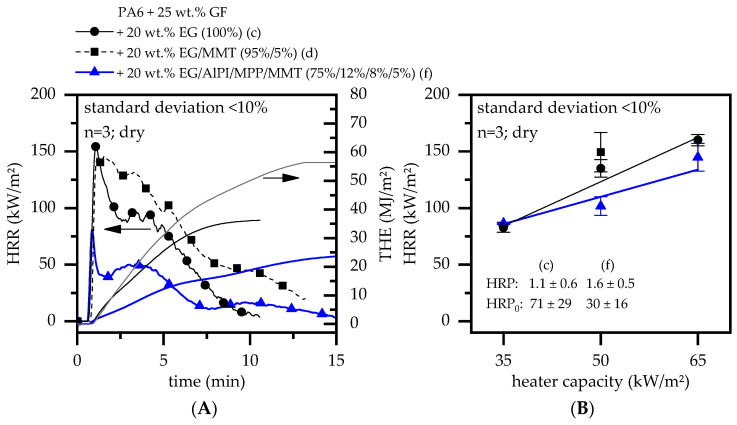
(**A**) Cone calorimeter results measured at 50 kW/m^2^. (**B**) Heat response parameters calculated from the cone calorimeter results at 35, 50, and 65 kW/m^2^.

The cone calorimeter results of all glass-fiber-reinforced formulations tested within this study are discussed in the following section. The results are presented in Figure 3 and Figure 4.

Non-modified glass-fiber-reinforced PA6 is usually characterized by a steep increase in the heat release rate (HRR) (Figure 3A). Since a large fraction of polymeric fuel had been burned by the time the peak heat release rate (pHRR) was reached, the subsequent steep HRR decrease could be attributed to a quick reduction in the pyrolysis gas flow rate. No plateau formation could be observed for the given sample thickness, indicating no stationary burning state. Thus, further acceleration of the heat development would likely occur for thicker samples. By incorporating MMT into the glass-fiber-reinforced PA6, a substantial change in the burning characteristics could be observed. Ignition occurred slightly earlier, followed by a steep increase in the HRR and a subsequent plateau formation. Since the heat development continuously decreased since then, decay of the pyrolysis gas evaporation could be assumed. As a result, the pHRR decreased by about 57% compared to the non-flame retardant PA6/GF.

This behavior remained constant for various external heater capacities applied. When comparing the heat release parameter (HRP), which was calculated from cone calorimeter tests conducted at heater capacities of 35, 50, and 65 kW/m^2^, MMT-modified samples showed substantially lower pHRR development (19 ± 0.3 versus 7.1 ± 1.3). Given that only 3 wt.% of the polymer was substituted by MMT, the decrease in the pHRR by 35% compared to PA6/GF is remarkable. Accordingly, independent of the fire scenario, PA6/GF equipped with MMT showed superior fire behavior. This observation is in good agreement with findings in other studies, such as [51,52,54]. Two principal phenomena have been attributed to the performance increase:

- Labyrinth effect: Nanodispersed MMT platelets form a labyrinth-like structure in the composite, increasing the material viscosity due to stronger material–particle interaction and extending the path of pyrolysis gases into the gas phase. As a consequence, the rate of gasification drops, which reduces the amount of available fuel and, thus, the burning rate. It is furthermore assumed that, for some polymeric systems, the formation of intermediate aromatic structures is favored. Thus, the labyrinth effect results in a prolonged meso-phase retention time, which increases the probability of (intermediate) char formation. Similar findings have been reported for many authors for various nano-scale systems. The relevant literature can be found in [55,56], although it is not limited to these references.
- During the cause of a fire, MMT starts to migrate to the burning surface and accumulates to increase the char yield. This enhances the barrier formation, which reduces heat re-radiation toward lower layers of non-decomposed polymer fractions. As a consequence, lower decomposition/evaporation rates limit the fuel supply and thus the heat development. Studies reporting similar observations can be found in [54,57], although they are not limited to these references.

When integrating 20 wt.% EG, EG/MMT, or EG/AlPi/MPP/MMT into glass-fiber-reinforced PA6, a substantial polymer fraction is substituted by the flame retardant additive system (Figure 4A). Hence, generally, lower heat development is to be expected. Since EG is the major flame retardant additive component providing improved thermal conductivity properties, heat can penetrate and dissipate more quickly over the entire cross-section. As the EG expansion and barrier formation proceed, the thermal isolation performance increases, counteracting the external heat penetration, lowering the heat penetration depth, and thus reducing heat development over time. Since barrier formation is a time-consuming process and residue formation in early burning stages needs some time to develop a sufficient thermal barrier, the heat release rate of formulations solely containing EG is relatively higher than that found for formulations additionally containing MMT or AlPi/MPP/MMT. By integrating MMT into the formulation, the EG barrier effect is combined with the previously described labyrinth effect. However, since the observable effect on the heat development is rather long-term and no change in the pHRR can be detected, the labyrinth effect is assumed to be abrogated by the expansion process. Hence, in formulations containing PA6/GF/EG/MMT, no synergistical effect could be observed for MMT integration.

By integrating AlPi/MPP in addition to EG/MMT into glass-fiber-reinforced PA6, the pHRR and average HRR are substantially reduced compared to EG and EG/MMT formulations. AlPi/MPP has been reported to react mostly in the mesophase, forming an aluminum phosphate residue [35,58]. When additionally combined with MMT, studies found a strong residue-solidifying effect [59], which could also be identified for formulations within this study. Furthermore, the decomposition mechanism of MPP partially evaporates melam and inert gases such as ammonia, providing an additional cooling and dilution effect [12]. The combination of long-term (EG) and short-term (AlPi/MPP) residue formation, pyrolysis gas stream reduction (MMT; labyrinth effect), improved residual stability (AlPi/MPP/MMT), and gas phase dilution (MPP) is thus found to work synergistically as a multi-material flame-retarding system for glass-fiber-reinforced PA6. The positive effect is also maintained over a set of different external heat capacities, resulting in a lower HRP of 1.1 (Figure 4B), indicating excellent flame retardancy performance with low sensitivity to environmental conditions.

The described results fit well with findings in previously published studies. MMT has been found to substantially decrease the burning rate in non-reinforced (e.g., [24,60]) and glass-fiber-reinforced (e.g., [54]) PA6, observable in a lower pHRR and prolonged burning times using cone calorimeter tests. Various combinations of PA6/AlPi/MMT (e.g., [7]) and PA6/AlPi/MPP/MMT (e.g., [59]) have been extensively investigated, particularly distinguishing phosphorus/MMT combinations to provide improved intumescent char formation. This effect is generally dedicated to a lower melt viscosity and nano-particle surface migration, accumulating and stabilizing residue formation on the polymer surface [61]. Glass-fiber-reinforced formulations containing EG and EG/AlPi/MPP have also been investigated (e.g., [23,25,35]). Results showed the lowest pHRR for PA6/GF formulations solely containing EG (<<100 kW/m^2^), whereas formulations based on EG/AlPi/MPP exceeded a pHRR rate of >100 kW/m^2^. Despite the apparently very low pHRR values, no UL-94 V0 classification could be achieved [25].

**Figure 5:**
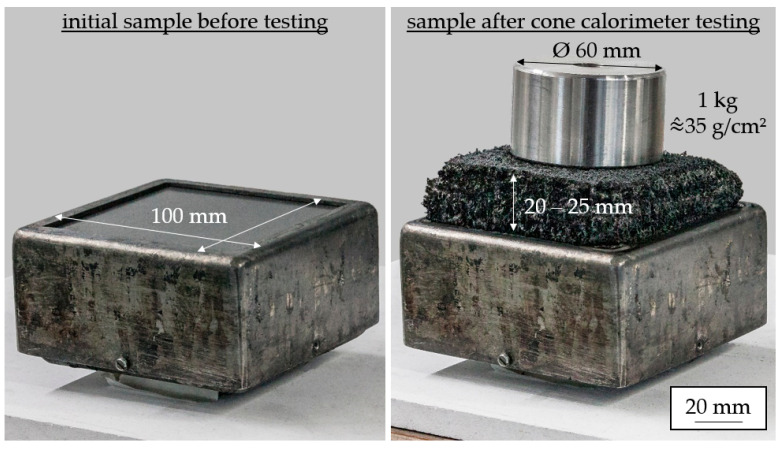
Initial sample (reference) and residue formation/stability after cone calorimeter testing.

The multi-material formulation (EG/AlPi/MPP/MMT) also showed superior properties regarding the residue stability. Figure 5 shows images of samples before and after cone calorimeter testing. The residue stability is visualized with a one kilogram weight, representing an area load of 35 g/cm^2^. No penetration of the weight into the residue surface could be identified.

Smoke production is another important property in fire critical applications. The general tendency of a burning material to generate smoke was evaluated via the key figure total smoke production (TSP) and that measured during cone calorimeter tests.

**Figure 6:**
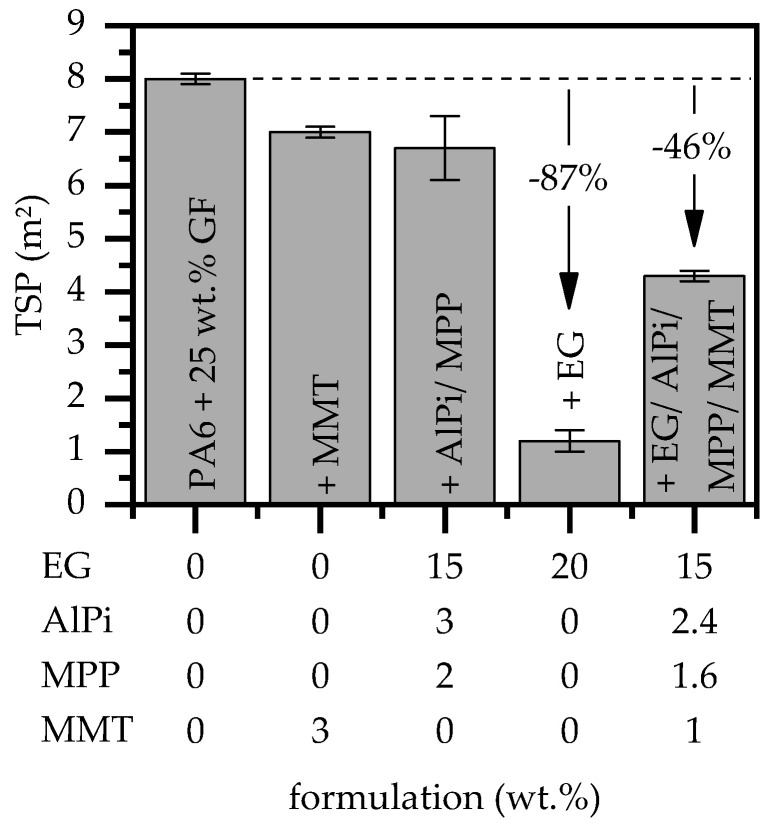
Total smoke production (TSP) measured via cone calorimeter testing with a heater capacity of 50 kW/m^2^.

The TSP measured for all of the tested formulations was in a range between 1.2 and 8 m^2^ (Figure 6). Formulations containing only AlPi/MPP or MMT compared to non-modified PA6/GF resulted in a slightly lower TSP between 6.5 and 7, which can be attributed to an overall reduction in the burning rate. The lowest value was observed for formulations containing only EG, since the flame retardancy mechanism is solely physical and provides an overall very low burning rate through voluminous residue formation. Through the integration of AlPi/MPP and MMT, the decomposition path is slightly disturbed, which leads to more incomplete combustion than that found solely for EG integration. As a consequence, the smoke production increases to 4.3 m^2^. All major cone calorimeter results are listed in Table 4.

### 3.3. Burning Behavior—UL-94 and LOI

**Figure 7:**
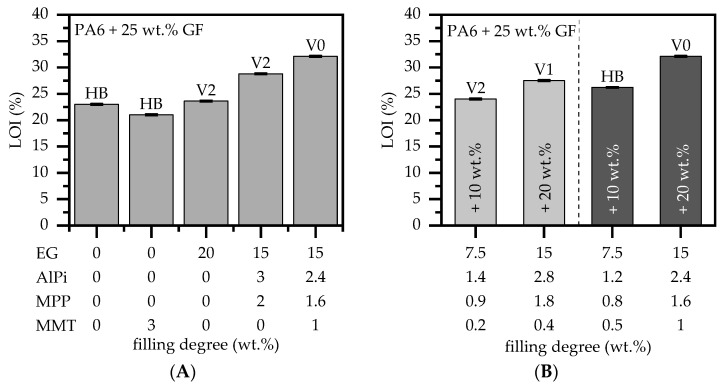
LOI and UL-94 testing results conducted for a sample thickness of 2 mm. (**A**) Results for singular and multi-material flame retardant additive integration. (**B**) Results of different filling degrees for two EG/AlPi/MPP/MMT formulations.

UL94 and LOI tests are used to evaluate self-extinguishing and ignitability properties, as well as those of polymeric materials. Figure 7 represents a selection of testing results conducted within this study.

In contrast to net PA6, which attains a V2 classification due to significant burn dripping, glass-fiber-reinforced PA6 did not achieve a UL-94 V classification. Burn dripping could not occur since the presence of glass fibers increased the melt viscosity. This also affected the LOI value, which reached 22% and was in good agreement with the literature values [62,63]. PA6/GF formulations containing 20 wt.% EG exhibited slightly better results in UL-94 (V2 classification) and LOI tests (24%). EG acts exclusively as a physical barrier, restricting the rate of pyrolysis gas that fuels the flame. EG is generally known to exhibit weaker performance in flammability tests because the barrier layer formed during the early burning stages takes some time to achieve sufficient effectiveness in order to stop the ignition process [25]. By integrating EG/AlPi/MPP as a multi-material system into PA6/GF formulations, the oxygen index could be improved to reach 28%. However, the UL-94 testing results did not demonstrate any improvement. This behavior aligns with the findings of a previously published study [25].

Contrary to the results discussed earlier in the cone calorimeter section, the integration of MMT nanocomposites into PA6/GF showed a deteriorating effect on the oxygen index, measured to be reaching 19%. No UL-94 classification could be achieved. This effect has also been reported in the literature [54,63]. Nanofillers have been investigated in various material systems and have been found to perform equally or worse in LOI and UL-94, but they perform substantially better in cone calorimeter tests. The effect is attributed to a change in melt viscosity, which alters the melt and burn dripping behavior. UL-94 and LOI fire tests are sensitive to melt dripping, since combustible fuel is physically removed from the burning area. The higher melt viscosity given through nanofiller integration suppresses a strong melt flow, negatively affecting UL-94 and LOI fire testing results [54,63]. However, when integrating MMT in the multi-material formulation PA6/GF/AlPi/MPP/MMT, a significant improvement in UL-94 and LOI fire testing results can be observed. Figure 7B shows the UL-94 and LOI fire testing results for two formulations, with filling degrees of 10 wt.% and 20 wt.%. Interestingly, formulations containing a lower MMT loading level (0.2 wt.%) perform better at a lower filling degree, but do not achieve a sufficient UL-94 V0 classification at a higher filling degree of 20 wt.%. Formulations containing 0.5 wt.% and 1 wt.%, on the other hand, do not provide a UL-94 classification for a filling degree of 10 wt.% but provide a UL-94 V0 classification when 20 wt.% of the multi-material mixture is added into the system. All data are summarized in Table 5.

### 3.4. Char Residue Analysis

**Figure 8:**
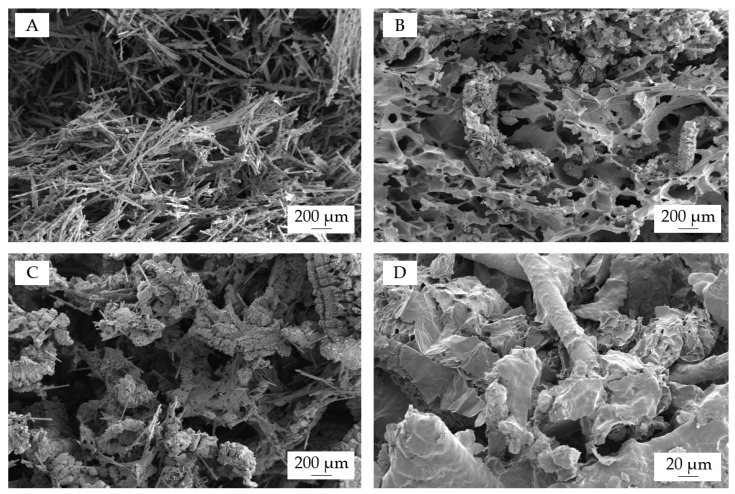
SEM analysis of char left after cone calorimeter testing. (**A**) PA6/GF/MMT, (**B**) PA6/GF/EG/MMT, and (**C**,**D**) PA6/GF/AlPi/MPP/MMT.

Char residue analysis was conducted using SEM analysis of the residual fractions left after cone calorimeter testing. Figure 8 shows a selection of images taken from various formulations. Residual fractions of PA6/GF/MMT can be described as a non-connected glass fiber network (Figure 8A). MMT could also be visually identified as agglomerated white powder fractions, yet it cannot be optically presented due to image resolution boundaries. Additionally, integrating EG into the system provides more voluminous sponge-like residue characteristics, whereas a combination of crosslinked char residue and residual polymer fractions acts as glue between the three-dimensional glass fiber network (Figure 8B). This is further improved via the addition of AlPi/MPP (Figure 8C,D). The aluminum phosphate residue, which is a result of AlPi/MPP decomposition, increases the amount of charring between the glass fibers and expanded graphite particles. This results in a more char-like structure, which provides superior structural stability, as previously shown in Figure 5.

## 4. Conclusions

This paper has investigated a multi-material flame retardant additive system based on EG, AlPi, MPP, and MMT in glass-fiber-reinforced PA6. This study was able to show that the sole integration of EG, while exhibiting excellent flame retardant properties in cone calorimeter tests, did not achieve a satisfactory UL-94 classification at a filler content of 20 wt%. By additionally adding AlPi, MPP, and MMT, a synergistic effect could be achieved, resulting in a stable V0 classification and good cone calorimeter properties. The synergistic effect was attributed to a combination of functional thermal barrier formation (EG), improved char formation (AlPi/MPP), and the labyrinth effect (MMT). Consequently, pyrolysis gas flow rates decreased, leading to lower flammability overall and heat development characteristics.

## Figures and Tables

**Table 1:** Polymers, fillers, and flame retardant additives used within this study.

Materials	Type/Code	Manufacturer
polyamide 6 (PA6)	B27E	BASF SE, Ludwigshafen
glass fiber (GF)	CS7920	Lanxess AG, Cologne
expandable graphite (EG)	GHL HT 270	LUH GmbH, Walluf
aluminum diethylphosphate (AlPi)	Exolit 1230	Clariant AG, Muttenz
melamine polyphosphate (MPP)	MP200	BASF SE, Ludwigshafen
organic montmorillonite (MMT)	MAX CT 4260	BYK-Chemie, Wesel

**Table 2:** Overview of all polymeric material recipes prepared and tested within this study.

Sample Code	PA6 wt.%	GF wt.%	EG wt.%	MMT wt.%	AlPi/MPP (3:2) wt.%
a	75	25	0.0	0	0
b	72	25	0.0	3	0
c	55	25	20.0	0	0
d	65	25	19	1	0
e	55	25	15.0	0	3.0/2.0
f	55	25	15.0	1.0	2.4/1.6
g	65	25	7.5	0.2	1.4/0.9
h	55	25	15.0	0.4	2.8/1.4
i	65	25	7.5	0.5	1.2/0.8

**Table 3:** TGA measurement summary of the most important key figures and activation energies calculated using the Ozawa–Flynn–Wall method, with TGA heating rates of 2.5, 5, and 10 K/min.

Sample Code	PA6 wt.%	GF wt.%	EG wt.%	MMT wt.%	AlPi/MPP (3:2) wt.%	T_99%_ Onset °C	Residue %	Activation Energy Ozawa
a	75	25	0	0	0.0	417.1	24.8	211
b	72	25	0	3	0.0	417.1	27.4	221
c	55	25	20	0	0.0	410.1	43.3	200
d	55	25	15	0	3.0/2.0	407.3	40.7	194
e	55	25	15	2	1.8/1.2	403.4	39.55	239

**Table 4:** Summary—fire testing results using cone calorimeter; 50 kW/m^2^.

Sample Code	PA6 wt.%	GF wt.%	EG wt.%	MMT wt.%	AlPi/MPP (3:2) wt.%	t_ign_ s	pHRR kW/m^2^	THE MJ/m^2^	MAHRE kW/m^2^	TSP m^2^
a	75	25	0	0	0.0	128 ± 4	531 ± 100	109 ± 10	235 ± 29	8.0 ± 0.0
b	72	25	0	3	0.0	112 ± 1	226 ± 8	68 ± 0	118 ± 8	7.0 ± 0.0
c	55	25	20	0	0.0	60 ± 1	134 ± 7	31 ± 5	66 ± 6	1.2 ± 0.2
d	55	25	15	0	3.0/2.0	56 ± 4	155 ± 6	35 ± 5	64 ± 3	4.3 ± 0.2
e	55	25	15	1	2.4/1.6	123 ± 2	102 ± 8	21 ± 4	33 ± 1	3.8 ± 0.2

**Table 5:** Summary—UL-94 and LOI testing results.

PA6 wt.%	GF wt.%	EG wt.%	MMT wt.%	AlPi/MPP (3:2) wt.%	UL-94 2 mm	t1 s	t2 s	C_ign_	LOI 2 mm %
75	25	0	0	0.0	HB—full burn to holder			yes	22.0 ± 0.2
72	25	0	3	0.0	HB—full burn to holder			yes	20.9 ± 0.2
55	25	20	0	0.0	V2	10 ± 2	8 ± 3	yes	36.0 ± 0.1
55	25	15	0	3.0/2.0	V2	6 ± 7	7 ± 3	yes	28.8 ± 0.2
55	25	15	1	2.6/1.4	V0	0 ± 0	0 ± 0	no	32.1 ± 0.2

C_ign_: cotton ignition.
